# Analysis of oral cancer carcinogens in repeatedly heated cooking oils

**DOI:** 10.1016/j.heliyon.2025.e41858

**Published:** 2025-01-14

**Authors:** Vidhant Krishna Nambiar, Vidya Mudliar, Inosi Salababa

**Affiliations:** a Department of Public Health, School of Dentistry and Oral Health, College of Medicine, Nursing and Health Sciences, Fiji National University, Suva, Fiji; b Department o*f* Oral Rehabilitation, School of Dentistry and Oral Health, College of Medicine, Nursing and Health Sciences, Fiji National University, Suva, Fiji; c Oral Surgery Unit, Dental Department, CWM Hospital, Ministry of Health and Medical Services, Fiji

**Keywords:** Oral cancer, Genotoxicity, 3-Mono chloropropanediolesters (3 MCPD), Glycidol, benzo[a]pyrene (BaP)

## Abstract

**Background:**

The consumption of fried food has assimilated itself as a part of food culture globally. Therefore, it is important to know the toxigenicity of cooking oils used when exposed to high temperatures. The incidence of oral cancer in recent years has been on the rise; ninety percent of the cancers present in the oral cavity are squamous cell carcinoma with multiple major contributing lifestyle factors such well as the presence of the human papilloma virus. Not all mechanisms of carcinogenesis are fully understood and are complex. Furthermore, most cooking oil manufacturers do not provide recommended cooking temperatures on their product labels. Instead, they typically advise storing oils away from direct sunlight and at room temperature, leaving consumers unaware of the safe usage lim-its during cooking.

**Objective:**

This study was conducted to analyze repeated cooking oils available in Suva, Fiji for harmful levels of genotoxic carcinogens. **Methodology**: Six types of cooking oils (soya bean oil, olive oil, mustard oil, coconut oil, canola oil and sunflower oil) were analyzed for the presence of genotoxic carcinogens. The test group (T0-3) of oils were heated to 190 °C and tested for the presence of carcinogens using Gas chromatography Mass Spectroscopy after 1.5 h at recommended temperature (T0), reheated to 190 °C and tested after 1 h (T1), reheated to 190 °C and tested after 3 h (T2) and reheated to 190 °C and tested after 6 h (T3). The control group of oils were not heated and stored at recommended temperature before testing.

**Results:**

The concentrations of Benzo[a]pyrene and Glycidol detected in the cooking oils tested were not significant, as they remained below 2 μg/kg. However, two measurable peaks in 3-monochloropropanediol (3-MCPD) were detected in olive oil (115.6 ng/ml) and Soya bean oil (117.2 ng/ml).

**Conclusion:**

Elevated 3-MCPD levels were found in Soya bean and olive reheated cooking oils exceeding tolerable daily intake levels and indicating potential health risks. Future research should evaluate the carcinogenic potential of cooking oils in real-world settings, such as fast-food establishments. This could inform public health policies on safer oil usage practices and raise consumer awareness about the risks of consuming foods cooked with overheated or reused oils.

## 1 Introduction

Oral cancer is the seventh most common type of cancer in humans, whilst constituting half of all head and neck cancers [1]. The incidence of oral cancer in recent years has been on the rise; ninety percent of the cancers present in the oral cavity are squamous cell carcinoma (SCC) with multiple major contributing lifestyle factors such as tobacco and alcohol use, betel quid chewing, as well as the presence of the human papilloma virus [2]. All mechanisms of carcinogenesis are not fully understood and are complex and it is important to analyze the most frequently used products that individuals are exposed to extrapolate potential risk assessments for humans.

Despite refining cooking oils for over a century to remove unwanted products, only recently have there been discoveries made of the presence of toxic compounds such as 3-monochloropropanediol esters (3 MCPA) and glycidol [3]. The compound 3MCPA is a known carcinogen dependent on the tolerability of daily intake. Studies have reported that various food constituents can affect or modify cancer cells [4]. Certain foods can also influence a person's genetic makeup and cause epigenetic cellular changes. Independent of the Deoxyribonucleic Acid (DNA) sequence itself, chromatic remodeling and changes in gene expression are referred to as "epigenetics” [5].

An abundance of biological reactions induced by the process of "frying," results in pro-duction of numerous chemical compounds. These degraded compounds may include free fatty acids (FFA), aldehydes, alkanes, 4-hydroxy nonenal, hydro-peroxide volatile compounds, and polymerized triglycerides. As the amount of heat applied to the oil increases significantly, more toxic chemicals and lipid peroxidation products are formed in the cooking oil. The degrees of alteration in the oil generally dictate the quality of oil for human consumption [6].

An earlier study has indicated that on average repeated cooking oils (RCO's) are used 3–6 times before being disposed as waste and its findings showed that there were six times as many total polycyclic aromatic hydrocarbons (PAHs) in RCO as there were in fresh cooking oil [7,8].

Earlier studies have found an association between frequent consumption of RCO's with various forms of cancers, including breast, lung, colorectal and prostate cancers [9,10]. However, not many studies are available that have analyzed the association between consumption of RCO's and oral malignancies. With oral cancer cases on the rise globally, RCO's needs to be analyzed as it has been reported to contain significantly high levels of process contaminants such as glycidyl esters (GE's) which have been proven to be oral cancer carcinogens [11]. The GEs break down during digestion releasing glycidol which is a genotoxic carcinogen and therefore all efforts should be made to minimize the quantity of GE in food as much as is practically practicable.

This is of grave importance to Fiji and the Pacific Island countries (PICS) as the most recent statistics of vegetable oil consumption per capita demonstrated an increase in con-sumption of Soya bean oil and olive oil from 2004 to 2018 [12]. The trend for the use of these oils is identical to Australia and New Zealand now where olive oil is largely the staple of oil in their diets [13]. Moreover, the Fiji Human Papilloma Virus and Related disease summary report depicted that oral cavity cancer rate is the highest incidence rate when it comes to head and neck cancers and a published media report from Fiji Times stated that, “Oral cancer is on the rise in Fiji with two to three or even more cases diagnosed per month” [14]. The rise in the number of oral malignancies and the lack of cases with no known risk factors raises red flags.

A study conducted on rats showed direct correlation of glycidol with oral squamous cell carcinoma (SCC), with an increased prevalence of squamous cell papilloma (SCP) of the mouth and tongue in female rats than male rats [15]. The carcinogenicity of glycidol is postulated to be due to genotoxic activity, which is probably a direct effect of the compound and not due to metabolic activation [16].

Varied cooking oils may have different genotoxic effects; hence the quality of the oil may be important. The 2015–2020 Dietary Guidelines for Americans also suggest that quality of cooking oils, rather than quantity, should receive more consideration. There is general agreement that after activating metabolically, polycyclic aromatic hydrocarbons (PAHs), which are indirect-acting carcinogens, become genotoxic and cause DNA damage, genetic instability, and the appearance of micronuclei (MN) in oral mucosa [17]. MN frequency is a sensitive precancerous marker for anticipating genotoxic outcomes like cancer and can effectively reflect the molecular dose of genotoxicity of micro pollutants. An earlier study showed that the bioactivation of benzo[a]pyrene (BaP), dibenz[a,h]anthracene (DBahA), benzo[b]fluoranthene (BbFA), and benzo[a]anthracene (BaA) towards the binding of cellular DNA in human oral epithelial cells were potent oral carcinogens [18]. This study therefore aimed to analyze the contents of repeated cooking oils available in Suva, Fiji, for harmful levels of genotoxic and known carcinogens.

## 2 Material and methods/experiment

### 2.1 Materials

This was an experimental study analyzing six types of cooking oils available in Suva, Fiji for the presence of genotoxic carcinogens. The selection of cooking oils for this study was evidence-based, relying on supermarket sales data, market surveys, and existing research to identify the most frequently used oils in Suva, Fiji. Sales reports from major retailers highlighted soybean, olive, mustard, coconut, canola, and sunflower oils as top-sellers, while surveys with consumers and retailers confirmed their widespread use in households and commercial cooking. Additionally, these oils reflect both traditional and modern dietary preferences, with coconut oil being a staple in Fijian cuisine and others catering to diverse culinary habits. This approach ensured the study's findings were relevant to the cooking practices of the local population (Table 1).

**Table 1:** Types of vegetable cooking oils.

Oils	Manufacturer	Composition
Soya bean oil	Punjas	Soya bean oil, vitamin A & D3
Olive oil	Basso	100 % pure refined olive oil
Mustard oil	Punjas	100 % pure mustard oil
Coconut oil	Fiji Coconut Millers	100 % pure coconut oil
Canola oil	Simply	100 % Canola oil
Sunflower oil	Simply	100 % Sunflower oil

#### 2.1.1 Sample preparation and testing

**Fig. 1:**
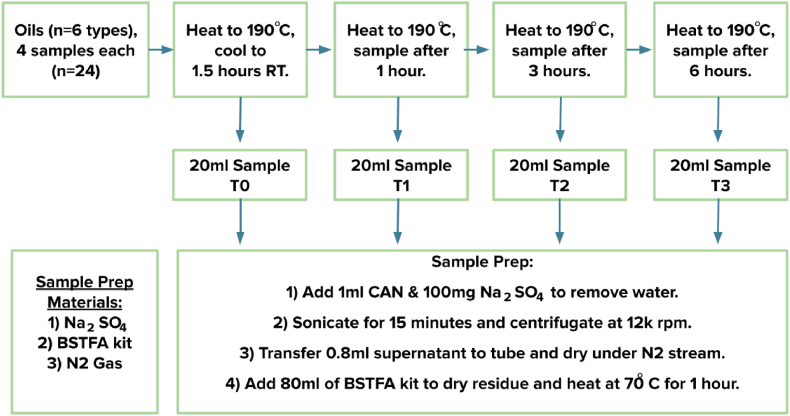
Sample preparation for test groups.

Each type of oil was divided into five groups of 20 ml each. Each oil sample was mixed with 1 mL of acetonitrile then with 100 mg of sodium sulphate (Na_2_SO_4_) to remove trace level water. The mixtures were then sonicated for 15 min and centrifuged at 12000 rpm for 10 min. Then 0.8 mL of the supernatant was transferred into a centrifuge tube and evaporated under a gentle nitrogen stream. BSTFA (Bis(trimethylsilyl)trifluoroacetamide) kit (80 μL) was added to the dry residue and then heated at 70 °C for 1 h to form thermos-stable trimethylsilyl derivatives (Fig. 1).

The control group of oils were not heated and stored at recommended temperature before testing. The test group of oils were heated to 190^◦^**C** and tested for the presence of carcinogens using Gas chromatography Mass Spectroscopy after 1.5 h at recommended temperature (T0), reheated to 190 °C and tested after 1 h (T1), reheated to 190 °C and tested after 3 h (T2) and reheated to 190 °C and tested after 6 h (T3).

### 2.2 Calibration of standards

The calibration curve for the determination of benzo[a]pyrene, glycidol and 3MCPD were constructed by plotting the peak areas from gas chromatography (GC) analysis against the corresponding concentrations of benzo[a]pyrene, glycidol and 3MCPD standards. The calibration curves R-Squared values for the three carcinogens were at an average of 0.99 which determined that the standard calibration curves were valid (Table 2).

**Table 2:** Calibration curve equation for standards withe R-squared values.

Standard	Calibration Curve Equation	R-Squared value
BaP	y = 22.530279x + 6323.237432	0.99909416
3-MCPD	y = 2.0800007x + 2232.691890	0.99267401
Glycidol	y3.066778e-006x2 + 0.148874x + 7621.107956	0.98966021

### 2.3 Analysis

**Fig. 2:**
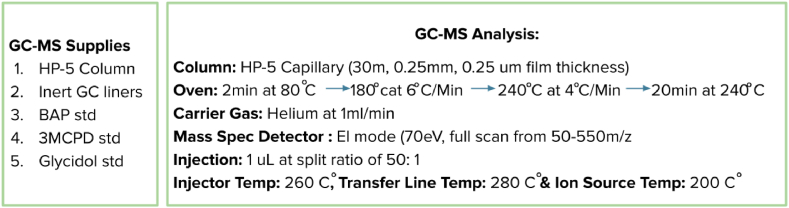
GC-MS analysis.

Gas chromatography-mass spectrum (GC-MS) (Teledyne Tekmar) analysis was performed using Agilent 6890N GC coupled with a 5975B MS detector. A HP-5 capillary column (30 m, 0.25 mm i.d., 0.25 μm film thickness) was used for chromatographic separation. All test samples were heated in an oven with a starting temperature of 80 °C for 2 min, increasing to 180 °C at 6 °C/min, and finally to 240 °C at 4 °C/min where temperature was maintained for 20 min. Helium was used as the carrier gas at a flow rate of 1 mL/min. The MS operated in EI mode (70 eV). All testing data was obtained using full scan mode from *m*/*z* 50 to 550. The temperature of the injector, the transfer line and the ion source were maintained at 260 °C, 280 °C and 200 °C respectively. One 1μL (μL) of derivatized samples was injected with a spilt ratio of 50:1 for MS detection (Fig. 2).

### 2.4 Data analysis

Mass spectral data from GC-MS profiling was extracted using Agilent data analysis software. Principal component analysis (PCA) and partial least squares-discriminant analysis (PLS-DA) was conducted using SIMCA-P+. Oil constitutes with variable importance in projection (VIP) above 1 was selected as potential markers. Statistical comparison of dis-criminatory markers between the various oils were carried out using *t*-test. The p-value below 0.05 was statistically significant. All markers were identified by searching against NIST (National Institute of Standards and Technology) library and further confirmed by using commercial standards.

## 3 Results and discussion

### 3.1 Benzo[a]pyrene

This study results did not show any significant concentrations of BaP as below the European Union (EU) maximum allowable limit for BaP (2 μg/kg) in oil and fat samples [17] (Table 3). The peaks for all tested oils were lower than the limit of detection from the calibration curve. According to the results from blank trace comparison, the interference that was present was not near the runtime range for the peaks of BaP.

**Table 3:** Concentration of Benzo[a]pyrene (ng/mL).

Oils	Control		T0		T1		T2		T3	
	Resp.	Final Conc. (ng/ml)	Resp.	Final Conc. (ng/ml)	Resp.	Final Conc. (ng/ml)	Resp.	Final Conc. (ng/ml)	Resp.	Final Conc. (ng/ml)
Canola	0	0	0	0	0	0	0	0	413.2085	0
Coconut	0	0	942.271	0	481.3905	0	370.062	0	271.598	0
Mustard	312.407	0	412.485	0	371.667	0	602.6085	0	219.3375	0
Olive	108.8095	0	0	0	0	0	195.032	0	159.2875	0
Soya bean	434.6595	0	387.686	0	996.872	0	0	0	0	0
Sunflower	195.167	0	193.6395	0	209.373	0	199.0875	0	376.253	0

### 3.2 3-MCPD

Two measurable peaks in 3-MCPD were detected at T3 for olive oil (115.6 ng/ml) and Soya bean oil (117.2 ng/ml). However, there were no significant peaks detected at T4 for either of the samples (Table 4).

**Table 4:** Concentration of 3-MCPD (ng/ml).

Oils	Control		T0		T1		T2		T3	
	Resp.	Final Conc. (ng/ml)	Resp.	Final Conc. (ng/ml)	Resp.	Final Conc. (ng/ml)	Resp.	Final Conc. (ng/ml)	Resp	Final Conc. (ng/ml)
Canola	0	0	0	0	0	0	0	0	0	0
Coconut	0	0	0	0	0	0	0	0	0	0
Mustard	0	0	0	0	0	0	0	0	0	0
Olive	0	0	0	0	0	0	171.9695∗	115.608∗	0	0
Soya bean	0	0	0	0	0	0	204.039∗	117.15∗	0	0
Sunflower	0	0	0	0	0	0	0	0	0	0

Soya bean oil (T2):

1. Mass conversion of oil from milligrams (mg) to kilograms (kg): 20 mg = 20/1000 = 0.02 kg
2. Total amount of 3- MCPD in micrograms (μg):

117 ng = 117/1000 = 0.117 μg (since 1 ng = 1/1000 μg)

3. Concentration in μg/kg:

0.117 μg/0.02 = **5.85** μ**g/kg**.

Olive oil (T2):

1 Mass conversion of oil from milligrams (mg) to kilograms (kg): 20 mg = 20/1000 = 0.02 kg
2. Total amount of 3-MCPD in micrograms (μg):115.6 ng = 115.6/1000 = 0.1156 μg
3. Concentration in μg/kg: 0.1156 μg/0.02 = **5.78** μ**g/kg**

### 3.3 Glycidol

Similarly, for glycidol, peaks for all tested oils were below the limit of detection for all resulting in the concentration being insignificant to be reported (Table 5).

**Table 5:** Concentration of glycidol (ng/ml).

Oils	Control		T0		T1		T2		T3	
	Resp.	Final Conc. (ng/ml)	Resp.	Final Conc. (ng/ml)	Resp.	Final Conc. (ng/ml)	Resp.	Final Conc. (ng/ml)	Resp.	Final Conc. (ng/ml)
**Canola**	123.9125	0	0	0	162.132	0	236.4495	0	317.8055	0
**Coconut**	255.1225	0	694.2405	0	172.303	0	148.611	0	410.327	0
**Mustard**	175.872	0	233.31	0	196.975	0	77.658	0	1035.44	0
**Olive**	135.255	0	0	0	196.825	0	0	0	260.7595	0
**Soya bean**	0	0	0	0	163.163	0	222.849	0	158.56	0
**Sunflower**	215.2705	0	182.055	0	0	0	176.739	0	296.626	0

### 3.4 Discussion

In this study, benzo[a]pyrene, glycidol, and 3-MCPD were determined in various cooking oils by constructing calibration curves and conducting sample runs to assess the presence and concentrations of these compounds. To ensure the accuracy of our results, blank trace comparisons were performed, and no significant issues were detected, affirming the reliability of the equipment and methodology used. However, we encountered challenges related to accurately detecting glycidol and benzo[a]pyrene at low concentrations due to their chemical properties and potential interference from other compounds in the oils.

In accordance with the European Food Safety Authority (EFSA) and the Joint FAO/WHO Expert Committee on Food Additives (JECFA), the tolerable daily intake levels of 3-MCPD (3-monochloropropane-1,2-diol) are established in micrograms per kilogram of body weight (μg/kg bw), with the evaluation setting the levels at 2.0 μg/kg bw and 4.0 μg/kg bw, respectively. These values represent the maximum amount of 3-MCPD that an individual can consume daily without adverse health effects, serving as important guidelines for regulatory authorities and food safety standards [17].

Measurable peaks of 3-MCPD were observed in olive oil (T2) and Soya bean oil (T2). Based on this toxicological data the potential risk of 3-MCPD towards human health was determined through risk assessment authorities such as the European food safety Authority (EFSA) and The Joint Expert Committee on Food Additives. A toxicological risk assessment based on EFSA and JECFA's findings indicates that exceeding the daily intake of 2 μg/kg bw has shown carcinogenic effects in rodent [18].

The International Agency for Research on Cancer (IARC) classifies 3-MCPD as a Group 2B carcinogen (possibly carcinogenic to humans) and glycidol as a Group 2A carcinogen (probably carcinogenic to humans) [19]. The tolerable daily intake (TDI) in this study was exceeded in both soybean oil (T2) and olive oil (T2), signaling potential health concerns related to reheated vegetable oils [19]. While similar studies have reported varied 3-MCPD levels in coconut oil, elevated concentrations were not reflected in this study, potentially due to limited refining processes for edible coconut oil in Fiji [18,20].

These natural variations in oil content and fatty acid profile can lead to inconsistent levels of contaminants like 3-MCPD, glycidol, and benzo[a]pyrene across different mustard oil samples or batches [21]. Similar variability can also occur in coconut oil due to factors like origin, cultivar, and processing methods. Mustard oil composition can vary significantly depending on factors like cultivar, origin, and processing methods. The oil content in mustard seeds ranges from 30 to 39 %, with PC-5 variety having the highest oil content at 39.26 %. Erucic acid is the major fatty acid, comprising 35–60.2 % of the oil composition, with PC-5 having the highest erucic acid content [22]. Differences in mustard oils from different countries have also been observed, with Chinese mustard oils containing unique compounds not found in oils from other regions [23].

The detection of glycidol in this study was lower than the level of significance. Similar results were also reported in a study conducted in Germany, which explored scenarios involving frequent consumption of frying fats with elevated levels of bound glycidol, resulting in a calculated margin of exposure (MOE) of 15,315. These results further emphasize the need for a follow-up study that would analyze reheated cooking oils that are exposed to food and fats found in most carbohydrates and food available at most fast-food chains as glycidyl esters adhere more intimately to these fats [24]. Since glycidol has been identified as a carcinogen affecting multiple sites (oral mucosa, brain glioma, thyroid gland follicular cell adenoma, leukemia, and mammary gland) its potency is consistent across all tissues when estimating the disease burden of total cancer. This raises serious concerns as glycidol is associated with the formation and decomposition of 3- and 2-MCPD. It forms monoesters with fatty acids (GE) during the refining of vegetable oils formed by the elimination of hydrochloric acid from MCPD monoesters that have a vicinal chlorohydrin structure. These monoesters are 3-MCPD that is esterified in the 1- or 2-position and 2-MCPD monoesters [25]. Glycidol's non-volatile nature and other compounds present in the sample may have presented challenges in the detection of glycidol within this study. As is the case with 3-MCPD, derivation methods and further scrutiny of column selection should be considered for future studies.

The analysis of reheated oils also revealed peaks corresponding to alpha-benzopyrene, a known carcinogen. Although these levels were below the limit of detection in our calibration curve, the findings prompt concerns regarding the potential health implications of prolonged exposure to trace levels of carcinogens.

Throughout this investigation, it is noteworthy that fresh vegetable cooking oil typically harbors fewer breakdown products, consequently yielding lower levels of benzo[a]pyrene and related compounds. Thus, the source and history of the cooking oil may be a crucial factor in determining the risk associated with oral exposure to benzo[a]pyrene. Benzo[a]pyrene is a well-established carcinogen, and its association with oral cancer has been extensively studied. Prolonged and frequent exposure to benzo[a]pyrene through the consumption of contaminated foods, particularly those cooked at high temperatures or with reused oils, pose a significant risk to oral health [26]. Despite falling below, the limit of detection (LOD) on the calibration curve, the identification of benzo[a]pyrene in reheated cooking oils raises potential health hazards. This apprehension is particularly salient when considering the oil's origin and historical usage.

This study not only stands as a commendable exploration of 3-MCPD in reheated oils but also sets the stage for follow-up research. The potential impact of fried food on glycidol and benzo[a]pyrene levels make it a valuable resource for designing comprehensive studies on the broader implications of cooking practices on public health.

## 4 Conclusion

Within the limitations of this study, several key findings emerged. Elevated levels of **3-monochloropropanediol (3-MCPD)** were detected in reheated soybean and olive oils, surpassing tolerable daily intake levels and indicating potential health risks. Minimal levels of **glycidol** and **benzo[a]pyrene** were also observed, underscoring the need for further research to understand their occurrence and potential impact.

A critical consideration in risk assessment is the real-world context in which these oils are used. While this study utilized fresh oils, it is likely that oils used in frying food may generate higher concentrations of glycidol and benzo[a]pyrene due to interactions with food particles and extended heating. This underscores the importance of integrating real-world cooking scenarios into future research to refine our understanding of health risks.

Future investigations should adopt a broader approach by analyzing oil samples collected from fast food restaurants and comparing them with fresh oils from these six brands. Such comparative studies would offer insights into the changes in contaminant levels resulting from actual cooking practices. This would enable more precise risk assessments and foster the development of evidence-based health recommendations.

## CRediT authorship contribution statement

**Vidhant Krishna Nambiar:** Writing – review & editing, Writing – original draft, Visualization, Validation, Supervision, Resources, Project administration, Methodology, Funding acquisition, Data curation, Conceptualization. **Vidya Mudliar:** Writing – review & editing, Writing – original draft, Visualization, Methodology, Funding acquisition, Formal analysis, Data curation. **Inosi Salababa:** Writing – review & editing.

## Data availability

Data will be made available upon request.

## Declaration of competing interest

The authors declare that they have no known competing financial interests or personal relationships that could have appeared to influence the work reported in this paper.
